# Durum Wheat at Risk in a Climate Change Scenario: The Carotenoid Content is Affected by Short Heat Waves

**DOI:** 10.1021/acs.jafc.4c05718

**Published:** 2024-09-05

**Authors:** María Dolores Requena-Ramírez, Cristina Rodríguez-Suárez, Dámaso Hornero-Méndez, Sergio G. Atienza

**Affiliations:** † Instituto de Agricultura Sostenible (IAS), Consejo Superior de Investigaciones Científicas (CSIC),Avda. Menéndez Pidal s/n. ,Córdoba 14004, Spain; ‡ Instituto de la Grasa (IG), CSIC, University Campus Pablo de Olavide, Building 46, Ctra. de Utrera, km 1, Sevilla 41013, Spain

**Keywords:** grain quality, heat stress, protein, thousand kernel weight, vitreousness

## Abstract

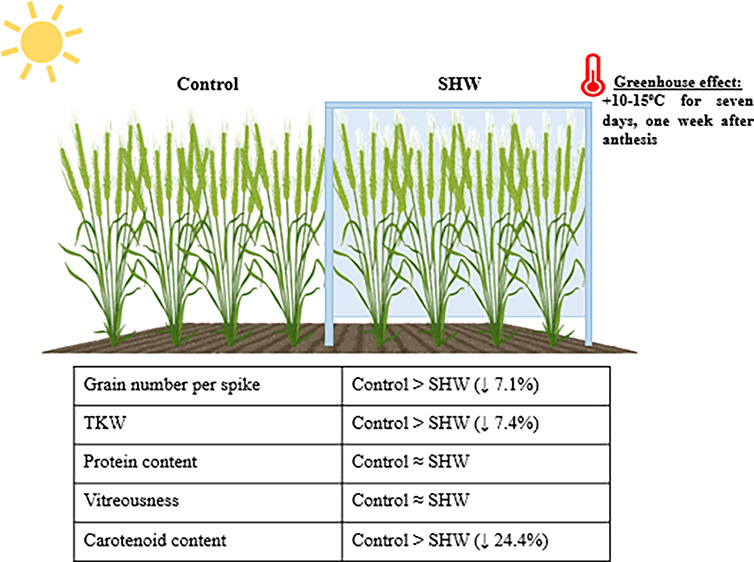

Short heat waves (SHW), defined as periods of several consecutive days with high temperatures above the developmental optimum, will become more frequent due to climate change. The impact of SHW on yield and yield-related parameters has received considerable interest, but their effects on grain quality remain poorly understood. We employed a simulation approach to investigate the impact of SHW on durum wheat quality over a 7 day period, starting 1 week after anthesis. During the SHW treatment, carried out using portable polyethylene tents, the temperature in the treated plots increased by 10–15 °C during daily hours. The SHW treatment reduced the number of grains per spike, thousand kernel weight, and total carotenoid content in grains in stressed plants in comparison to control plants. However, no differences in the protein content or percentage of vitreous grains were observed. The behavior of individual carotenoids in response to SHW appears to differ, suggesting a differential change in the balance between β,ε- and β,β-branches of the carotenoid biosynthetic pathway as a consequence of SHW-induced stress. The present study highlights the importance of developing efficient breeding strategies for reduced sensitivities to heat stress. Such strategies should not only prioritize yield but also encompass grain quality.

## Introduction

Durum wheat [(*Triticum turgidum* L. var. *durum* (Desf.) Husn.] is the 10th most important cereal in the world, with an average annual production of 40 million Tm, representing 5% of total wheat.^1^ Production of durum wheat grains meeting the high standards for end-use suitability is limited to a few regions worldwide, including the Mediterranean Basin.^1^ Climate change will have a significant impact on both crop productivity and quality. The Intergovernmental Panel on Climate Change (IPCC) has projected a global warming trend of 0.3–1.7 °C by 2100,^2^ which is a major concern for agriculture in the medium- to long-term. In addition, short heat waves (SHW), periods of several consecutive days with high temperatures above the developmental optimum, will be also more frequent. The frequency of high temperatures around the anthesis period is predicted to increase in Europe.^3^ In wheat, the occurrence of very high temperatures at anthesis has a negative effect on yield by reducing spike fertility and grain filling period, impairing grain filling.^3,4^

The exponential increase in the number of publications on this topic in recent decades indicates a growing concern about the consequences of heat stress due to climate change on wheat performance.^5^ In particular, heat waves will become more frequent and intense.^6,7^ A clear example of the impact of SHW during anthesis and early grain filling on durum wheat productivity was evidenced in Italy.^8^ Analysis of the incidence of SHW during these phenological phases (around May to June) showed severe reductions in durum wheat yields, with higher yield reductions coinciding with stronger SHW events.^8^ It has been demonstrated that both pre-anthesis and post-anthesis heat stress lead to yield penalties, although yield is more sensitive to pre-anthesis heat stress.^9^ The reduction in the number of grains per spike and the effects on spike initiation, floral organ differentiation, and sporogenesis are the main causes of yield losses due to pre-anthesis heat stress.^10^ At anthesis, heat stress increases floret abortion.^11^ In addition, high temperatures during the period from anthesis to grain maturity reduce grain yield by shortening the time available for the plant to acquire resources.^10^

SHW must be addressed as a serious concern in Mediterranean environments, as they will occur with increasing frequency in the future.^7^ In addition to this, it is well documented that high-temperature regimes lead to a reduction in grain weight, which is detrimental to grain quality.

The effect of SHW on the yield and yield-related parameters has received considerable interest in recent years. However, little is known about the potential effects of SHW on grain quality, although high temperatures affect the biosynthesis and accumulation of many grain compounds. In wheat, high temperatures lead to changes in the composition of the gluten proteins^12^ and moderate heat stress has been shown to negatively affect bread-making properties due to an increase in extractable HMW glutenin (high-molecular weight glutenin).^13^ It can also affect dough properties, starch content, and essential amino acid levels (for a comprehensive review, see ref (14)). Grain yellowness is a crucial trait in durum wheat, influencing the quality of pasta and couscous. This character is of paramount importance because color is the first attribute assessed by consumers when determining food quality, conditioning its acceptability and consumption.^15^ Consequently, worldwide durum wheat breeding programs focus on yellow grain color as a key quality trait.^16,17^ Carotenoids are the pigments responsible for the yellow color of grains of durum wheat and other cereals belonging to the Triticeae tribe. Lutein is the major carotenoid in the endosperm of durum wheat and related cereals, representing 90–95% of the total carotenoid content.^17^ It is worth noting that the intake of lutein and zeaxanthin is associated with a lower risk of developing age-related macular degeneration in humans,^18^ which has been linked to the depletion of macular carotenoids.^19^ In addition, carotenoids are natural antioxidants that provide important health benefits including provitamin A activity, the inhibition of carcinogenesis, and the reduction of the risk of developing cardiovascular and other chronic diseases.^20−22^ The importance of carotenoids in relation to the end-use characteristics of durum wheat has led to the elucidation of the genetic bases for carotenoid biosynthesis and degradation, the development of marker-assisted selection programs, and the implementation of diverse methodologies for the determination of these compounds for breeding purposes (for a review, see ref (17)). However, there is a lack of knowledge regarding the impact of heat stress on the carotenoid content in wheat grains. In a previous experiment, we demonstrated that the carotenoid content was reduced by increasing temperature in common wheat, despite the absence of heat stress conditions.^23^ Consequently, further research on this topic under field conditions is needed, particularly in view of the challenges and threats posed by climate change in Mediterranean areas.

The objective of this study was to examine the impact of SHW on the quality traits of durum wheat grains, including the carotenoid content and profile, protein content and vitreousness, and yield-related traits such as a thousand kernel weight and seed set on spikes. This was done to ascertain the extent to which durum wheat is susceptible to this stress under Mediterranean conditions.

## Materials and Methods

### Plant Material

Four durum wheat elite varieties were selected for the present study: “Don Ricardo” (Agroovegetal, year of registration 2008); “Don Ortega” (Agroovegetal, year of registration 2018), “Athoris” (Limagrain Iberica, S.A. year of registration 2011); and “Amílcar” (Bayer Cropscience, year of registration 2002). Field experiments were carried out at “Finca Alameda del Obispo” (Córdoba, Spain) under irrigated and well-fertilized conditions. The experiments were conducted using a randomized block design with two replications. The main plot size was 3 × 1.2 m (six rows separated by 20 cm), from which two subplots of the same size were established for control and treatment conditions. A seeding rate of 350 seeds/m^2^ was used. The post-anthesis heat stress was applied using portable tents to generate a greenhouse effect (adapted from ref (24)). One week after anthesis, the structures (1.5 × 1.5 × 1.5 m) were mounted on the field trials and covered with a transparent polyethylene film (125 μm). The lower ends of the four sides of the cages (25 cm height) were not covered to facilitate gas exchange. The SHW stress was maintained for a period of 1 week. The temperature inside and outside the cages was monitored using data loggers (EBI 20-T1, Ebro, Germany). Solar radiation MJ·m^–2^ d^–1^ and ambient temperature were obtained from the meteorological station located near the experimental field at IAS-CSIC (Córdoba, Spain).

### Sampling, Yield-Related Traits, and Quality Parameter Determination

At heading, all the spikes at the same physiological stage were labeled. At maturity, all labeled spikes were harvested, threshed, and used for quality (carotenoids, protein content, and vitreousness) and thousand kernel weight (TKW) determinations. The protein content (%) was estimated using a LECO Elemental Analyzer (LECO Macro CN828, Leco Corporation) for nitrogen determination. The protein content was calculated as follows: protein (%) = nitrogen (%) × 6.25. Vitreousness (%) was visually characterized using a Grobecker cutting tool in an external commercial service provided by Sehicor, S.A. (Córdoba, Spain).

The grain set per spike was determined from 10 labeled spikes, according to the spike position, considering the basal spikelet as position 1 and with continuous numbering up to the terminal spikelet.

### Carotenoid Analysis

Carotenoids were extracted and analyzed as described in ref (25). The grain sample (1 g) was placed into a 25 mL stainless-steel grinding jar together with two stainless-steel balls (15 mm diameter) and 6 mL of acetone containing 0.1% BHT (w/v). Samples were ground by using an oscillating ball mill Retsch Model MM400 (Retsch, Haan, Germany) at 25 Hz for 1 min. The resulting slurry was collected into a 15 mL polypropylene centrifuge tube and centrifuged at 4500*g* (5 min, 4 °C). The supernatant was transferred to a 15 mL polypropylene centrifuge tube, and the solvent was evaporated with a nitrogen stream. The dry extract was dissolved in 0.5 mL of acetone (HPLC-grade) and stored at −30 °C until use.

Carotenoid pigments were analyzed by HPLC by using a reversed-phase C18 column (200 mm × 4.6 mm i.d., 3 μm, Mediterranea SEA18; Teknokroma, Barcelona, Spain). A binary-gradient elution profile, composed of acetone (A) and deionized water (B), was used: Initial conditions (75% A; 25% B) increase linearly to 95% A in 10 min, then hold 95% A for 7 min and raises to 100% A in 3 min, and then maintained constant (100% A) for 3 min. The initial conditions were restored in 5 min. The flow rate was 1.0 mL/min, and the column was maintained at 25 °C. A diode-array detector was used for UV-visible spectrophotometric detection at 450 nm, and the online carotenoid spectra were acquired in the 330–650 nm wavelength range. Calibration curves were prepared with pure carotenoid standards (lutein, zeaxanthin, and β-carotene), and the chromatograms were integrated at 450 nm for quantification. The concentration of (*Z*)-isomers of lutein was determined using the calibration curve for (*all-E*)-lutein. Analyses were performed in duplicate and on the same day that the extracts were prepared. Data are expressed as μg/g fresh weight (μg/g fw).

### Statistical Analyses

Analyses of variance were performed using Statistix version 10.0 (analytical Software, Tallahassee, FL, USA). The use of *p* values was reported as continuous quantities following the recommendations in ref (26). Figures were obtained using the GGally package in RStudio v2024.04.1 Build 748.

## Results and Discussion

### Effectiveness of the Short Heat Wave (SHW) Treatment

The growing cycle was comprised from November 22nd 2022 (sowing) to May 24th 2023 (harvesting). The four varieties selected for this work are well adapted to Spanish conditions. Indeed, “Athoris”, “Amílcar”, and “Don Ricardo” represented almost 50% of the total certified seed produced in Spain during the 2022–2023 season (https://www.mapa.gob.es/es/agricultura/estadisticas/estadisticas-semillas.aspx), with relative contributions of 21.9, 17.4, and 8.3%, respectively. “Don Ortega” is a relatively new variety, representing less than 1% of the certified seed market in Spain.

**Figure 1:**
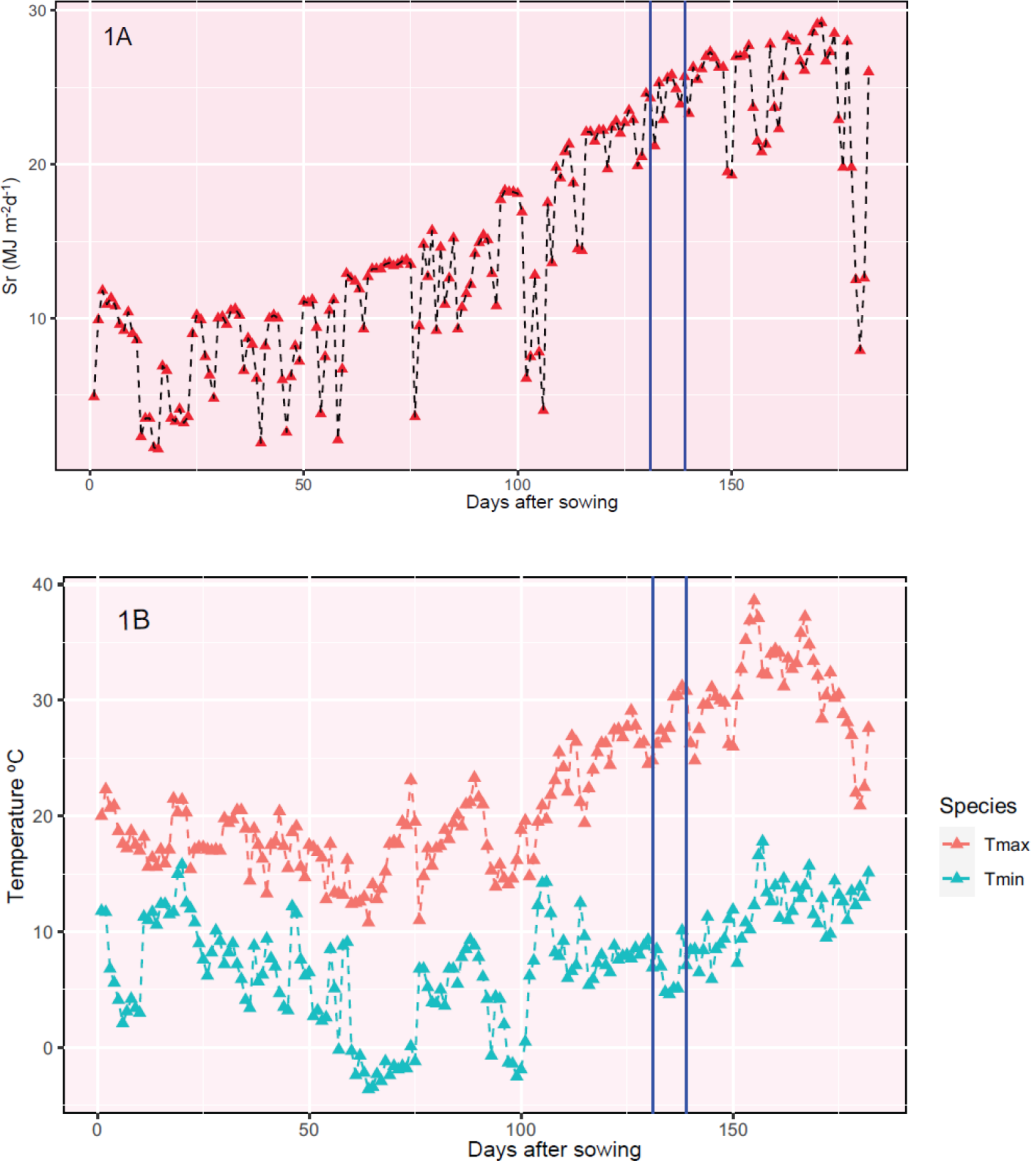
Environmental data during the growing cycle over the 2022–2023 season in Córdoba (Spain). (A). Solar radiation. (B). Daily temperatures. Vertical lines show when the SHW treatment was imposed. Sr = solar radiation; Tmax = maximum temperature; Tmin = minimum temperature.

Durum wheat varieties flowered within the same day, with the exception of “Athoris”, which flowered 1 day later. The SHW stress treatment was applied 1 week after anthesis, from the third to the 10th of April for “Amílcar”, “Don Ortega”, and “Don Ricardo” and from the 4th to 11th of April for “Athoris”. Solar radiation and maximum and minimum temperatures during the growing cycle are shown in Figure 1.

**Figure 2:**
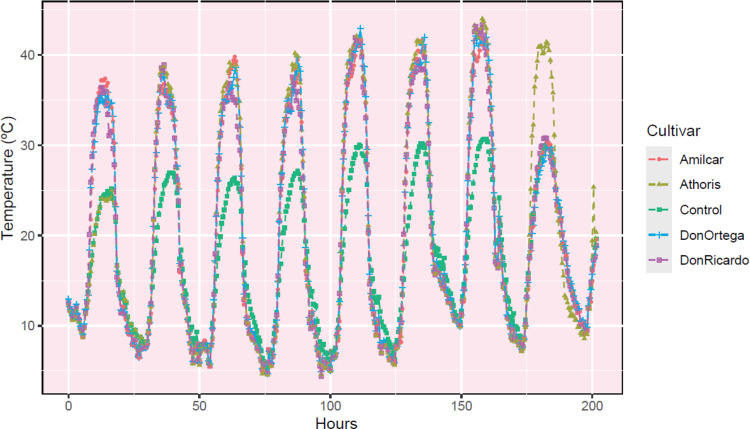
Hourly temperature dynamics of the SHW treatment for each variety compared to the average untreated controls.

During the SHW stress treatment, all days were sunny with solar radiation between 25 and 30 MJ·m^–2^ d^–1^ and the maximum ambient temperature was between 25 and 30 °C. This resulted in an effective SHW treatment with temperature increases between 10 and 15 °C and maximum temperatures around 40 °C in the treated plots (Figure 2). Similar studies conducted in northern Spain have reported milder SHW stress with increases in temperature in SHW plots of 5–10 °C,^9,24,27^ up to 8 °C,^27^ or up to 10 °C.^24^ The higher greenhouse effect observed in our experiment could be explained, at least partly, by the fact that we use a thicker polyethylene film (125 μm) compared with the film (100 μm) used in the above-mentioned works.

An important consideration of this work is that the SHW treatment was carried out under field conditions, and it resulted in an increase in air temperature to values that are expected to be reached in the future in our region due to climate change. Successful scaling up of results from the laboratory to the field is critical for effective use by plant breeders and agronomists, and eventual adoption by farmers.^28^ The polyethylene tent methodology has been proven reliable.^5,9,24^ Although no approach is perfect in reflecting the field conditions, this methodology provides a much closer picture of real conditions than experiments with isolated plants grown in pots. A potential concern has recently been raised, suggesting that if high humidity is reached inside the polyethylene tents, then the cooling capacity of the plants could be affected by reducing the transpiration rate, resulting in a slightly more intense SHW treatment.^9^

### Impact of SHW in Yield and Quality Traits

#### Grain Number per Spike

**Figure 3:**
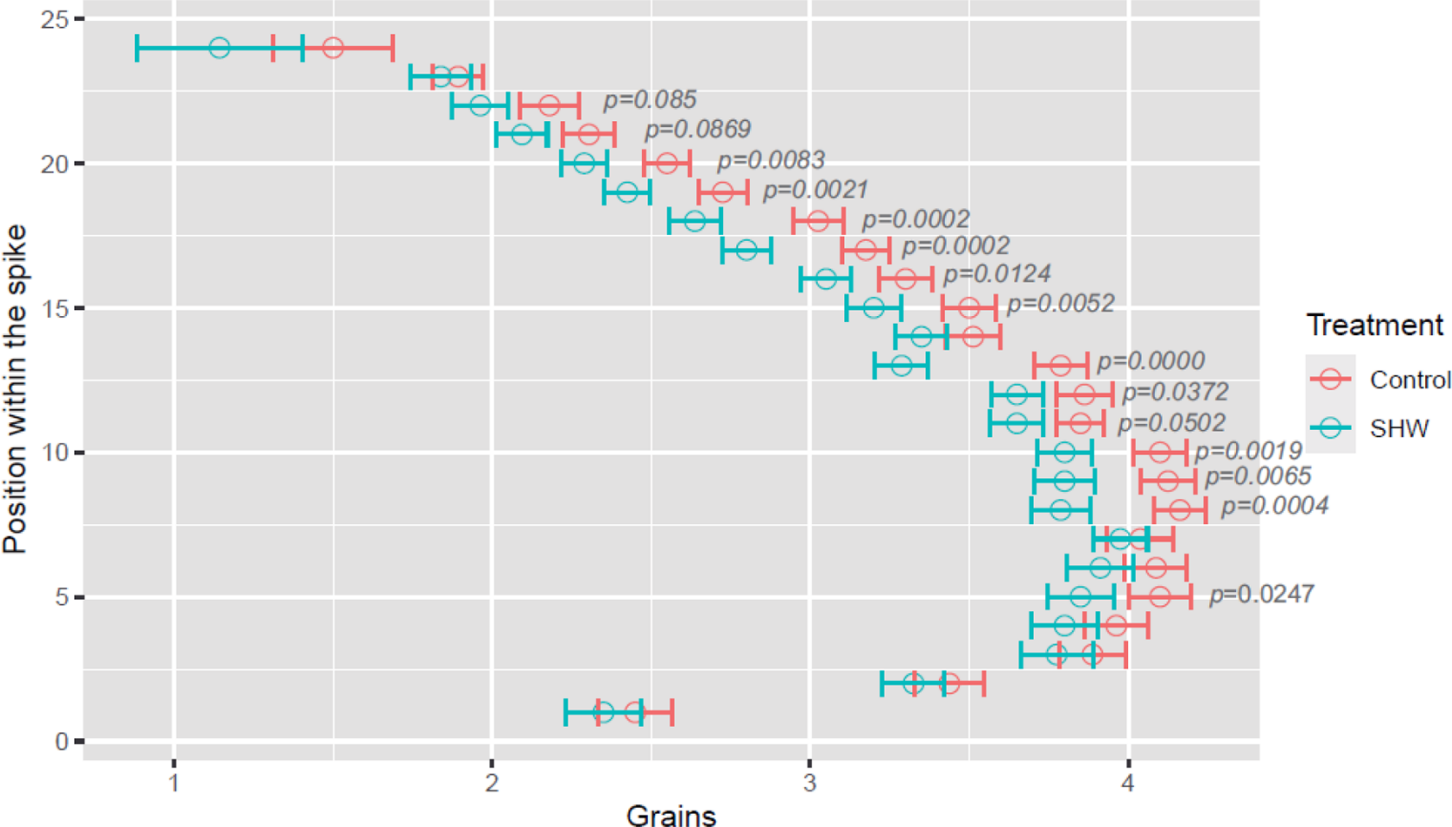
Effect of postanthesis SHW treatment on the number of grains per spikelet. Positions of each spikelet in the spike are numbered considering the bottom of the spike as position 1. For each position, the mean value of the 10 main spikes ± std. error is shown. For each spikelet, statistical significance between stress and control treatments is shown when *p* < 0.1.

The SHW treatment affected the grain number per spike (Table 1). Indeed, the number of grains per spike decreased by 6.9% between plants under stressed and control conditions (70.5 in SHW-treated vs 75.8 control, *p* = 0.026) with differences of grain set along the complete spike length (Figure 3). “Athoris” was the most sensitive variety to SHW with a 12.3% reduction, while “Don Ortega” was the least affected variety with a decrease of only 3.8% (Table 2).

**Table 1:** Effect of SHW Stress on Yield-Related and Quality Traits

treatment^a^	grains/spike	TKW (g)	protein (%)	vitreousness (%)
control	75.8 ± 2.7 a	51.2 ± 2.1 a	12.66 ± 0.13 a	93.9 ± 1.6 a
SHW	70.5 ± 3.4 b	47.4 ± 2.2 b	12.95 ± 0.18 a	93.0 ± 1.3 a
%reduction	6.9%	7.4%		

a Data are mean ± SE. SHW: short heat wave; % reduction = [100 – (SHW/control) × 100], only shown for traits with significant differences after Tukey’s HSD test). TKW: thousand kernel weight, expressed at 12% grain humidity. For each trait, values with the same letter are not significantly different at *p* < 0.05 as determined by Tukey’s HSD test. Difference Tukey’s HSD test *p* = 0.05.

**Table 2:** Differences between Genotypes for Yield-Related and Quality Traits

cultivar^a^	treatment	grains/spike	TKW (g)	protein (%)	vitreousness (%)
Amílcar	control	76.9 ± 1.26 bc	50.9 ± 0.09 ab	13.5 ± 0.25 ab	97.2 ± 1.65 a
Athoris	control	65.2 ± 1.75 d	54.6 ± 2.04 a	13.1 ± 0.04 abc	92.0 ± 2.25 a
Don Ortega	control	84.6 ± 1.62 a	42.3 ± 0.91 bc	11.8 ± 0.16 c	90.3 ± 2.18 a
Don Ricardo	control	76.5 ± 1.95 bc	56.8 ± 0.49 a	13.0 ± 0.22 ab	96.0 ± 0.77 a
Amílcar	SHW	72.3 ± 2 cd	46.9 ± 1.40 abc	12.8 ± 0.18 abc	92.3 ± 1.46 a
Athoris	SHW	57.2 ± 1.59 e	50.2 ± 0.26 ab	13.3 ± 0.13 a	94.2 ± 2.39 a
Don Ortega	SHW	81.3 ± 1.88 ab	39.2 ± 0.80 c	12.1 ± 0.28 bc	88.5 ± 1.66 a
Don Ricardo	SHW	70.9 ± 1.78 cd	53.2 ± 4.63 a	13.6 ± 0.49 a	97.0 ± 0.68 a

a Data are mean ± SE; TKW: thousand kernel weight; expressed at 12% grain humidity. For each trait, values with the same letter are not significantly different at *p* < 0.05 as determined by Tukey’s HSD test. Difference Tukey’s HSD test *p* = 0.05.

Floret fertility could not be affected by the SHW treatment as it was applied 1 week after anthesis. Therefore, the reduction in the grain number in the SHW stressed plants indicates grain abortion. It is widely accepted that the grain number is mainly determined before anthesis. Heat stress at this stage reduces the number of fertile florets, which significantly affects grain yield due to the reduced grain number.^29^ In addition, even if pre-anthesis SHW treatment does not result in a noticeable effect on floret death, it can still cause a considerable grain abortion rate.^9^ Indeed, these authors reported a grain abortion rate of 30–55%, with the cultivar with larger grains exhibiting a more pronounced response to the abiotic stress, as heat stress increased the lability of the fertile flowers to set a grain.^9^ Our results indicate that the post-anthesis SHW can also cause yield penalties by reducing the number of grains per spike. Similar results were observed in the cultivar “Karl92” when subjected to a 1 day heat stress of 38 °C.^30^ Heat stress causes dehydration, increased photorespiration, and reduced CO_2_ assimilation during grain filling.^10^ All of these factors are likely contributing to the reduction in the number of grains per spike, as shown in Figure 3.

#### Thousand Kernel Weight (TKW)

The TKW was found to be reduced in the SHW plots compared to the untreated plots (47.4 vs 51.2, respectively, *p* = 0.021), representing a 7.4% reduction due to SHW (Table 1). “Athoris” was also the most sensitive variety with an 8.15% reduction in TWK due to SHW stress, “Don Ricardo” being the less affected variety with only a 6.33% reduction (Table 2). The reduction in grain weight due to heat stress during grain filling has been well documented in previous studies. Back in the 1990s, Wardlaw and Wrigley^11^ provided an overview highlighting the main results presented at a specialized workshop on “heat tolerance in temperate cereals”, where it had already been shown that heat stress reduces grain size and weight. Heat stress reduces the grain filling period, which cannot be compensated by an increase in grain filling rate,^10^ resulting in a reduction in grain weight. The response of grain weight to temperature is genotype-dependent.^10^ In our case, the four varieties showed a similar behavior (with reductions in TKW between 6.33 and 8.15%) since they are varieties with good adaptation to our Mediterranean conditions, where high temperatures are common during grain filling.

#### Protein Content and Vitreousness

Our data did not show a noticeable effect on the grain protein content in response to the SHW treatment (12.95% in SHW vs 12.66% in control plants, *p =* 0.0926). Similarly, heat stress applied 1 week after anthesis for a period of 5 days also had no effect on the protein content.^31^ However, heat stress of 10 days or more applied 1 week after anthesis had a negative effect on the protein content.^31^ Therefore, longer SHW stresses in the field could affect the protein content. In addition, Wardlaw et al.^32^ reported a reduction in the ratio of high-molecular-weight to low-molecular-weight protein due to heat shock in common wheat, although no changes in grain protein were observed after heat shock treatment. In durum wheat, the protein content is more important than protein quality in pasta cooking.^33^ Medium and strong gluten varieties have advantages over weak gluten varieties for pasta firmness.^33^ Given that our experiments did not show any appreciable effects on protein quantity, it seems unlikely that the SHW stress could have affected gluten strength and hence pasta firmness, but we cannot completely rule out this possibility.

The vitreousness of the grain is important in milling, as vitreous grains give a higher yield of coarse semolina than starchy grains. A fully vitreous kernel requires sufficient protein content.^34^ No differences in the percentage of vitreous grains were observed between treatments (Table 1), which is consistent with the lack of pronounced differences in the protein content.

#### Carotenoid Content

The typical carotenoid profile of wheat grains was found in the SHW-treated and control plants. Lutein accounted for 92% of the carotenoid content in both control and SHW treatments, with minor amounts of other carotenoids, including zeaxanthin, β-carotene, and α-carotene (Table 3). “Athoris” and “Don Ortega” grains showed a higher grain carotenoid content than “Amílcar” and “Don Ricardo” under control conditions (Table 4). Accordingly, both cultivars had higher levels of lutein than “Don Ricardo” and “Amílcar”, although they behaved differently with respect to the minor carotenoids. The higher and lower levels of zeaxanthin were observed in “Don Ortega” and “Athoris”, respectively (0.192 vs 0.150 μg/g), while the opposite was observed for α-carotene (0.017 vs 0.046 μg/g in “Don Ortega” and “Athoris”, respectively). A similar trend was observed in SHW-treated plants. The total carotenoid content in SHW-treated plants was 24.4% lower than in control plants (2.22 μg/g in SHW-treated vs 2.94 μg/g in control plants). In a similar manner, the individual carotenoid levels were lower in grains from SHW-treated plants. As the levels of α-carotene and β-carotene were very low in both treatments, these data were not further considered in the discussion. The overall reduction in carotenoid levels, without any marked change in the carotenoid profile, suggests a general attenuation of the carotenogenic process in response to the abiotic stress imposed. These changes may be related to the role of carotenoids as radical scavengers^35^ and as precursors for the synthesis of some phytohormones such as abscisic acid (ABA)^22,36^ and strigolactones,^37,38^ which are key regulators of growth, development, and stress responses in plants. In addition, the response of plants to biotic and abiotic stress is characterized by the production of volatile terpenes, which provide a way for plants to communicate with other neighboring plants and animals (such as herbivores and insects) by sending attracting or deterring signals.^39^ The biosynthesis of the volatile terpenes uses geranylgeranyl pyrophosphate, which is also the general precursor of plant carotenoids (C40 tetraterpenoids), probably affecting the flux of the precursor to the carotenogenic pathway under stress conditions. The plant carotenoid pathway is divided into the β,ε-branch, which produces α-carotene and lutein, and the β,β-branch, leading to the synthesis of β-carotene and zeaxanthin, which are the precursors for strigolactone and abscisic acid (ABA) synthesis, respectively. As deducted from the decrease in the ratio β,ε/β,β (12.14 in SHW vs 13.40 in control), SHW may be exerting a differential effect on the two carotenogenic branches favoring the β,β-branch. In fact, lutein and zeaxanthin, the major representatives of the β,ε- and β,β-branch, respectively, appeared to behave differently as a result of SHW since the lutein level in SHW-treated plants was 24.9% lower than in controls, whereas the decrease was 15.1% for zeaxanthin. Further work will need to be done to investigate the insight into the SHW impact on carotenogenesis and other related pathways.

**Table 3:** Effect of SHW Stress on the Total and Individual Carotenoid Content Expressed in μg/g Fresh Weight

treatment^a^	Zeax^b^	Lut^c^	αCar^d^	βCar^e^	TC^f^	ratio β,ε/β,β^g^
control	0.177 ± 0.01 a	2.711 ± 0.18 a	0.023 ± 0 a	0.027 ± 0 a	2.938 ± 0.18 a	13.57 ± 0.99 a
SHW	0.150 ± 0 b	2.036 ± 0.2 b	0.016 ± 0 b	0.019 ± 0 b	2.220 ± 0.21b	11.92 ± 0.95 b
%reduction	15.1%	24.9%	33.0%	29.7%	24.4%	12.15%

a Data are mean ± SE. SHW: short heat wave; %reduction = [100 – (SHW/control) × 100].

b Zeax = (*all-E*)-zeaxanthin.

c Lut = lutein ((*all-E*)-lutein + (*Z*)*-*lutein isomers).

d αCar = (*all-E*)-α-carotene.

e βCar = (*all-E*)-β-carotene.

f TC = total carotenoids.

g Ratio β,ε/β,β = (Lut + αCar)/(Zeax + βCar). For each carotenoid compound, values with the same letter are not significantly different at *p* < 0.05 as determined by Tukey’s HSD test.

**Table 4:** Differences between Genotypes for the Total and Individual Carotenoid Content Expressed in μg/g Fresh Weight

genotype	treatment^a^	Zeax^b^	Lut^c^	αCar^d^	βCar^e^	TC^f^	ratio β,ε/β,β^g^
Amílcar	control	0.186 ± 0.01 a	2.061 ± 0.11 bcd	0.010 ± 0 d	0.026 ± 0 abc	2.284 ± 0.11 bc	9.8 c
Athoris	control	0.150 ± 0.01 c	3.314 ± 0.08 a	0.046 ± 0 a	0.033 ± 0 a	3.542 ± 0.09 a	18.4 a
Don Ortega	control	0.192 ± 0.01 a	3.473 ± 0.13 a	0.017 ± 0 cd	0.025 ± 0 abc	3.707 ± 0.14 a	16.1 ab
Don Ricardo	control	0.178 ± 0.01 ab	2.000 ± 0.09 bcd	0.021 ± 0 c	0.024 ± 0 bcd	2.217 ± 0.10 bc	10.0 c
Amílcar	SHW	0.156 ± 0.01 bc	1.691 ± 0.07 cd	0.007 ± 0 d	0.019 ± 0 cde	1.873 ± 0.08 bc	9.7c
Athoris	SHW	0.142 ± 0.01 c	2.673 ± 0.41 ab	0.035 ± 0 b	0.027 ± 0 ab	2.877 ± 0.43 ab	15.8 ab
Don Ortega	SHW	0.165 ± 0.01 abc	2.569 ± 0.40 abc	0.010 ± 0 d	0.016 ± 0 de	2.760 ± 0.40 ab	14.1 b
Don Ricardo	SHW	0.137 ± 0.00 c	1.210 ± 0.07 d	0.010 ± 0 d	0.014 ± 0 e	1.371 ± 0.07 c	8.1 c

a SHW: short heat wave.

b Zeax = (*all-E*)-zeaxanthin.

c Lut = Lutein ((*all-E*)-lutein + (*Z*)-lutein isomers).

d αCar = (*all-E*)-α-carotene.

e βCar = (*all-E*)-β-carotene.

f TC = total carotenoids.

g Ratio β,ε/β,β = (Lut + αCar)/(Zeax + βCar). For each carotenoid compound, values with the same letter are not significantly different at *p* < 0.05, determined by Tukey’s HSD test.

Previous experiments on wheat leaves have demonstrated the reduction of the total carotenoid content due to heat stress.^40^ Indeed, these authors reported that the carotenoid content was reduced during heat stress and subsequently during the recovery phase, provided that heat stress was maintained for at least 6 days. Our results are consistent with these findings. In fact, although the SHW stress was terminated well before the end of the grain-filling phase, the carotenoid content of the stressed plots could not be recovered, resulting in a lower total carotenoid at maturity. These results suggest that the carotenoid pool cannot be replenished during grain filling after an SHW of at least 7 days, as used in this work. Previous results from our group^23^ showed that the total carotenoid content was reduced by temperature, but in that work, the temperature differences between treatments were maintained throughout the grain-filling phase, and therefore, the results are not comparable.

Our results also showed a genotype-dependent behavior. “Athoris” and “Amílcar” showed the lowest reduction in the total carotenoid content due to SHW. These varieties had a total carotenoid penalty of about 18% due to SHW (2.877 μg/g in SHW vs 3.542 μg/g in control, “Athoris”; 1.873 μg/g in SHW vs 2.284 μg/g in control, “Amílcar”) (Table 4). “Don Ricardo” showed the highest reduction in the total carotenoid content (38.2%), and “Don Ortega” showed an intermediate reduction (25.5%) (Table 4). Both “Amílcar” and “Don Ricardo” had similar total carotenoid contents under control conditions (Table 4), but the carotenoid decrease in “Don Ricardo” was twice that of “Amílcar”. This suggests that the reduction in the carotenoid levels due to SHW is genotype-dependent and not related to the total carotenoid content. Indeed, “Athoris” has 1.6 times more carotenoids than “Amílcar” under control conditions, but they undergo the same suppression due to SHW. The genotype-dependent carotenoid content reduction due to heat stress has also been reported in flag leaves.^41^ These authors observed genotype-dependent changes in the total carotenoid content (increase, decrease, or no variation).^41^ The marked differences in the total carotenoid content after SHW found in our work highlight the importance of selecting genotypes less sensitive to SHW stress to ensure grain quality. In this context, it is crucial to understand the biochemical and molecular pathways influenced by SHW stress to identify the genetic bases behind this difference and to provide an efficient selection to identify genotypes less sensitive in relation to carotenoid downregulation.

In conclusion, SHW reduced the number of grains per spike and TKW in durum wheat, but no noticeable effects were observed on the grain protein content and vitreousness. The significantly lower levels of carotenoid contents in grains of plants grown under SHW stress indicate that grain quality needs to be considered when addressing the effects of climate change on wheat production. These results, together with the differences between genotypes, highlight the importance of developing efficient breeding strategies to select genotypes with less sensitivity to heat stress, which should focus not only on yield but also on grain quality. SHW stress appears to differentially affect the β,ε- and β,β-branches of the carotenoid pathway, although further research is needed to confirm this point.

## ABBREVIATIONS

αCar: (*all-E*)-α-carotene
βCar: (*all-E*)-β-carotene
BHT: butylated hydroxytoluene
HSD: honestly significant difference
Lut: lutein ((*all-E*)-Lutein + (*Z*)-lutein isomers)
SHW: short heat wave
TC: total carotenoids
TKW: thousand kernel weight
Zeax: (*all-E*)-zeaxanthin
